# Frontier and hotspots in the role of sentinel lymph node in endometrial cancer based on bibliometric analysis

**DOI:** 10.3389/fonc.2025.1585931

**Published:** 2025-09-01

**Authors:** Di-Ying Li, Yi Yang, Hai-Hui Xie, Yin Shao, Su Fang, Jin-Ling Zhang

**Affiliations:** ^1^ Department of Gynecology, Shenzhen People’s Hospital (The Second Clinical Medical College, Jinan University, The First Affiliated Hospital, South University of Science and Technology), Shenzhen, Guangdong, China; ^2^ Department of Gynecology, Shenzhen Futian District Maternity and Child Healthcare Hospital, Shenzhen, Guangdong, China

**Keywords:** endometrial cancer, sentinel lymph node, bibliometric analysis, VOSviewer, bibliometrix R

## Abstract

The role of sentinel lymph nodes (SLN) in endometrial cancer remains controversial. Our study is dedicated to employing bibliometric methods to explore the correlation between endometrial cancer and SLN. We aim to statistically analyze the existing literature on sentinel lymph nodes in the field of endometrial cancer research and explore future research trends and hotspots. Global literature on the role of SLN in endometrial cancer published from 1900 to the present in the Web of Science core database was searched. Cited articles were focused on by extracting information such as country, journal, keywords, institution and author. Descriptive statistics and visual analysis were performed using VOSviewer and R package. A total of 545 articles were screened, most of which were from USA (159 articles). Based on Bradford Law, Gynecologic Oncology and International Journal of Gynecological Cancer were core journals in this research field. Abu-Rustum Nadeem R from Mem Sloan Kettering Cancer Center was most productive author. The current research focus was endometrial cancer, SLN, lymphadenectomy, biopsy, indocyanine green (ICG). The complication, guidelines, outcomes and survival were the frontier of current research. Researchers in different countries have paid extensive attention to the role of SLN in the treatment of endometrial cancer, and have made breakthroughs in this field. SLN mapping has a promising prospect for the recurrence and prognosis of patients with endometrial cancer. More clinical research is needed to discover the role of SLN in the future.

## 1 Introduction

Endometrial cancer ranks as the sixth most common malignancy among women, with a striking 417,000 new cases reported worldwide in 2020 (1). Approximately 67% of patients are diagnosed at an early stage, with a 5-year overall survival (OS) rate of 81% for this group (2). Around 10–15% of cases present as advanced disease, and among those with distant metastases, the 5-year survival rate falls sharply to 16.8% (3, 4). Remains the primary treatment for early-stage endometrial cancer, typically involving total hysterectomy and bilateral salpingo-oophorectomy (BSO) as the standard surgical procedure (5). Assessment of lymph nodes during staging and the application of postoperative adjuvant therapy are both central to therapeutic success. In early-stage disease, pelvic lymphadenectomy has not been shown to provide benefit in long-term survival or recurrence prevention (6). Consequently, identifying and treating sentinel lymph nodes (SLN) plays a significant role in the clinical management of endometrial cancer.

The SLN represents the first site of lymphatic metastasis and serves as an initial barrier to the dissemination of tumor cells. It is now widely employed in managing breast cancer and head and neck malignancies, and is also applicable in melanoma, colorectal cancer, and cervical cancer (7–10). According to the National Comprehensive Cancer Network (NCCN) guidelines, pelvic lymph-node evaluation, potentially including paraaortic lymph-node dissection, is advised when high-risk features such as deep myometrial invasion or high-grade histology are present. For patients with intermediate- and high-risk endometrial cancer, ESGO recommends systematic lymph node dissection (11). Postoperative complication rates are higher in the pelvic lymph node dissection group compared to the non-dissection group (*p*=0.001), yet 5-year disease-free survival and overall survival rates do not differ significantly between the two cohorts (12). SLN mapping is now recommended over extensive lymphadenectomy in patients whose disease appears limited to the uterus (13). European guidelines indicate that SLN resection offers advantages over systematic lymph node removal for individuals at low to intermediate risk, and SLN metastasis has been linked to prognosis (14). SLN mapping has proven effective as a substitute for pelvic and para-aortic lymphadenectomy, lowering the incidence of complications such as lymphedema and lymphocyst formation (15). The negative predictive value of SLN biopsy stands at 95%, suggesting that when the SLN is cancer-free, the likelihood of metastasis in other lymph nodes is very low. While lymph node involvement remains a significant prognostic factor, it does not always drive treatment decisions (16, 17). Nevertheless, the role of SLN in endometrial cancer is not yet fully understood. Additional studies are required to determine its influence on therapeutic strategies and clinical outcomes.

The body of literature on SLN mapping in endometrial cancer has expanded steadily in recent years, yet no single publication has definitively established its role. Although global interest in SLN research for endometrial cancer continues to rise, pinpointing key studies through manual search remains difficult given the volume of available publications. Bibliometric analysis, which applies statistical methods to evaluate research output within a field, has proven useful for measuring the scientific influence of publications across disciplines (18). Despite this, a focused bibliometric assessment dedicated to the role of SLN in endometrial cancer has not yet been conducted. This study addresses that gap by examining the current research landscape on SLN application in endometrial cancer through a range of bibliometric techniques (19).

## 2 Methods

### 2.1 Data source

**Figure 1:**
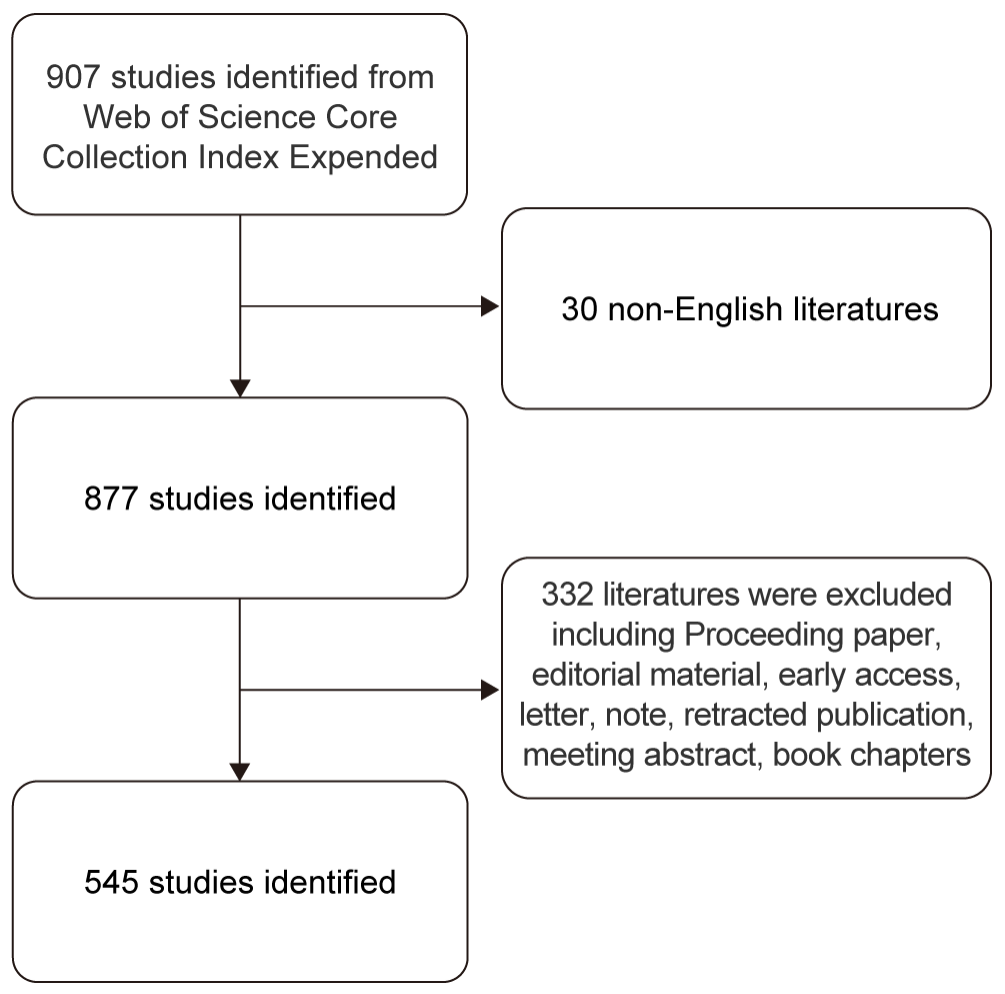
Flow chat of search strategy.

The literature search was conducted on April 27, 2024, using the Science Citation Index Expanded in the Web of Science Core Collection. The search employed the following MeSH terms: endometrial cancer and sentinel lymph node. A topic search covered abstracts, article titles, author keywords, and Keywords Plus. Keywords Plus are phrases extracted from the titles of referenced articles (20). To refine the results, the search terms were applied to the title (TI), abstract (AB), and author keywords (AK) fields in the advanced search mode, aiming for greater accuracy (21). Only English-language articles and reviews were included. Proceedings papers, editorial materials, early access articles, letters, notes, retracted publications, meeting abstracts, book chapters, and duplicate articles were excluded. A total of 907 records were initially identified, of which 362 were excluded based on the inclusion and exclusion criteria. The final dataset consisted of 545 articles, which were exported for analysis (**Figure 1**). Two independent authors verified the records for consistency.

### 2.2 Analysis methods

This study employed two computer-assisted tools to manage and analyze a large volume of bibliographic data: VOSviewer (version 1.6.20) and the R package “bibliometrix” (version 4.3.3) within RStudio (version 2023.12.1.0).

VOSviewer, a Java-based bibliometric tool developed by Van Eck and Waltman in the Netherlands and released in 2009 (22), is particularly effective in extracting structured data from a large corpus of academic publications (23). It groups related nodes into clusters, with same-colored nodes indicating stronger associations (19). The software also supports overlay visualizations, where node color, distance, and size represent temporal distribution and interrelationships across a two-dimensional space (24). In this study, VOSviewer was used to generate visual analyses of keywords, authors, countries/regions, institutions, and journals. Each circular node stands for an item such as a keyword, author, journal, or institution. Thicker lines between nodes signify a stronger degree of collaboration or co-citation between items (25–27).

The R package “bibliometrix” provides a set of functions for conducting bibliometric analysis. It is widely used for examining global publication trends and for applying Bradford’s Law, which describes how articles are distributed among journals in a given field (28).

## 3 Results

### 3.1 Annual publication trend

**Figure 2:**
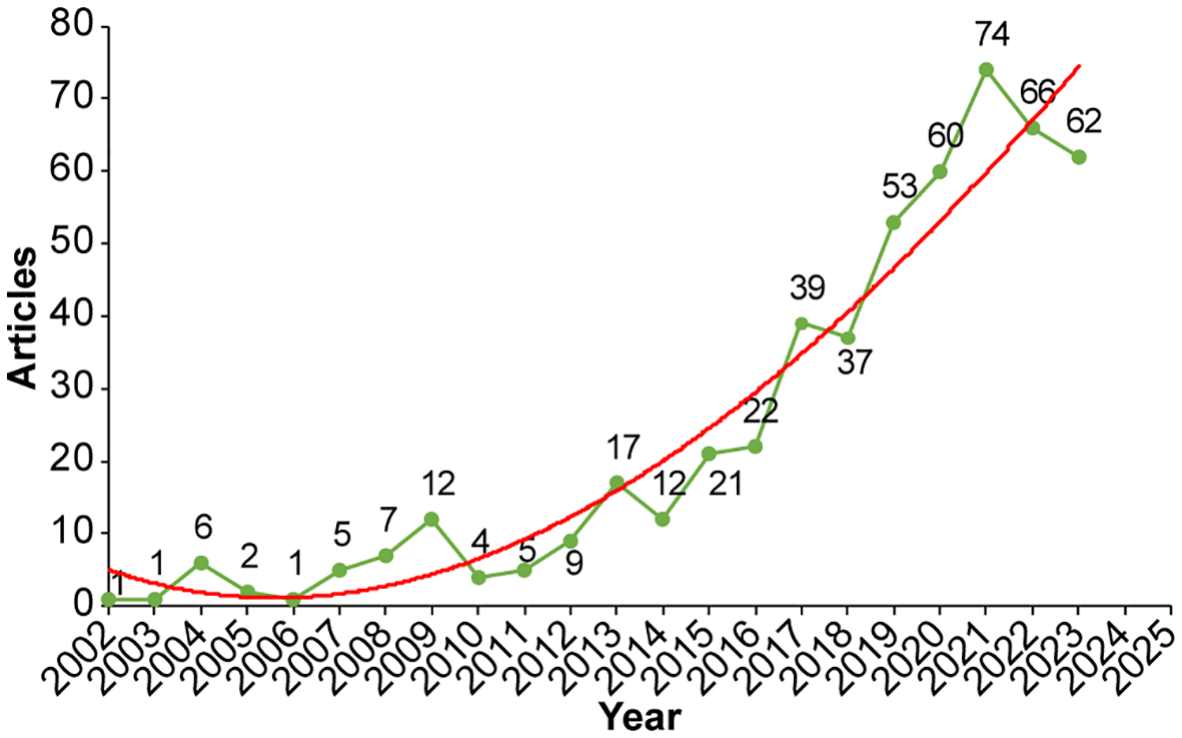
The annual number and trend of published articles.

A total of 545 articles on SLN mapping in endometrial cancer were identified, showing a clear upward trend in annual publication volume over recent years. The earliest publications date back to 2002, with a gradual increase between 2002 and 2014, during which the yearly output ranged from 0 to 17 articles (**Figure 2**). From 2015 onward, the number of publications began rising rapidly, reaching a peak of 74 articles in 2021. Based on the trajectory of the trend line, the number of annual publications is projected to reach approximately 133 by 2030.

### 3.2 Country/region analysis

**Figure 3:**
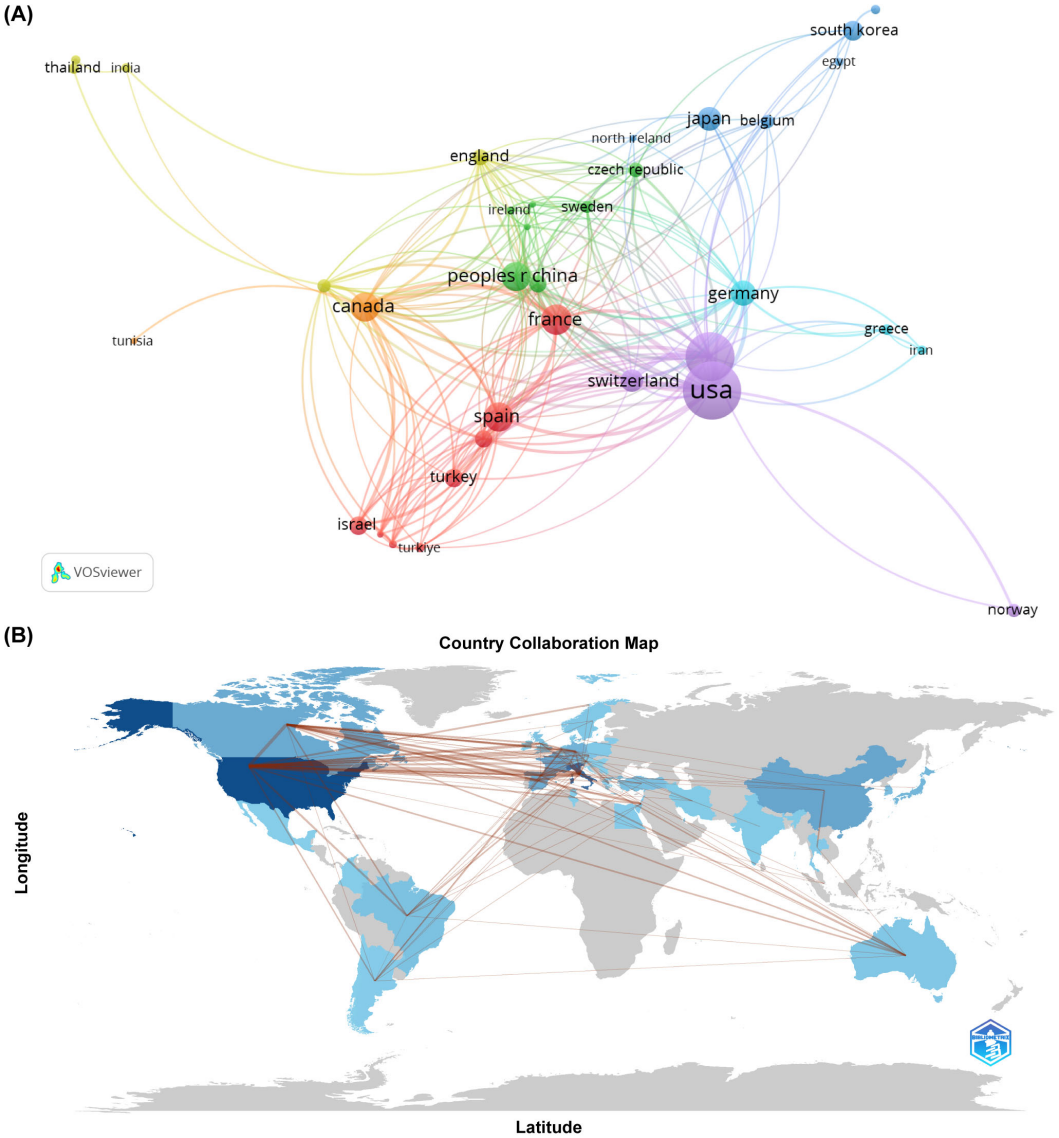
**(A) **The cooperation networks between countries/regions. **(B)** Visualized country collaboration map.

In terms of geographical contribution, 48 countries or regions have published work on SLN in endometrial cancer. The United States led with 159 publications, amassing a total of 2060 citations, an average citation rate of 37.4, and an H-index of 40. Italy and France followed, with 45 and 42 publications respectively (**Table 1**). Collaboration patterns (**Figure 3A**) showed that the United States, France, Canada, and China had established strong research ties with other countries. At the continental level (**Figure 3B**), North America and Europe were leading in both output and collaborative activity. South America, Australia, and East Asia followed, while Africa had the lowest publication count and the fewest collaborative connections.

**Table 1:** Top 10 countries/regions of published articles.

Rank	Country	Documents	Citation	Average citation/Publication	H-index
1	USA	159	2060	37.4	40
2	ITALY	114	1141	20.64	28
3	FRANCE	45	880	30.31	19
4	CANADA	42	1242	44.76	20
5	CHINA	42	231	7.52	10
6	SPAIN	39	480	16.74	15
7	GERMANY	32	462	18.16	13
8	JAPAN	28	404	18.96	12
9	SWITZERLAND	24	401	25.08	12
10	SOUTH KOREA	20	386	419	11

### 3.3 Institutional analysis

**Figure 4:**
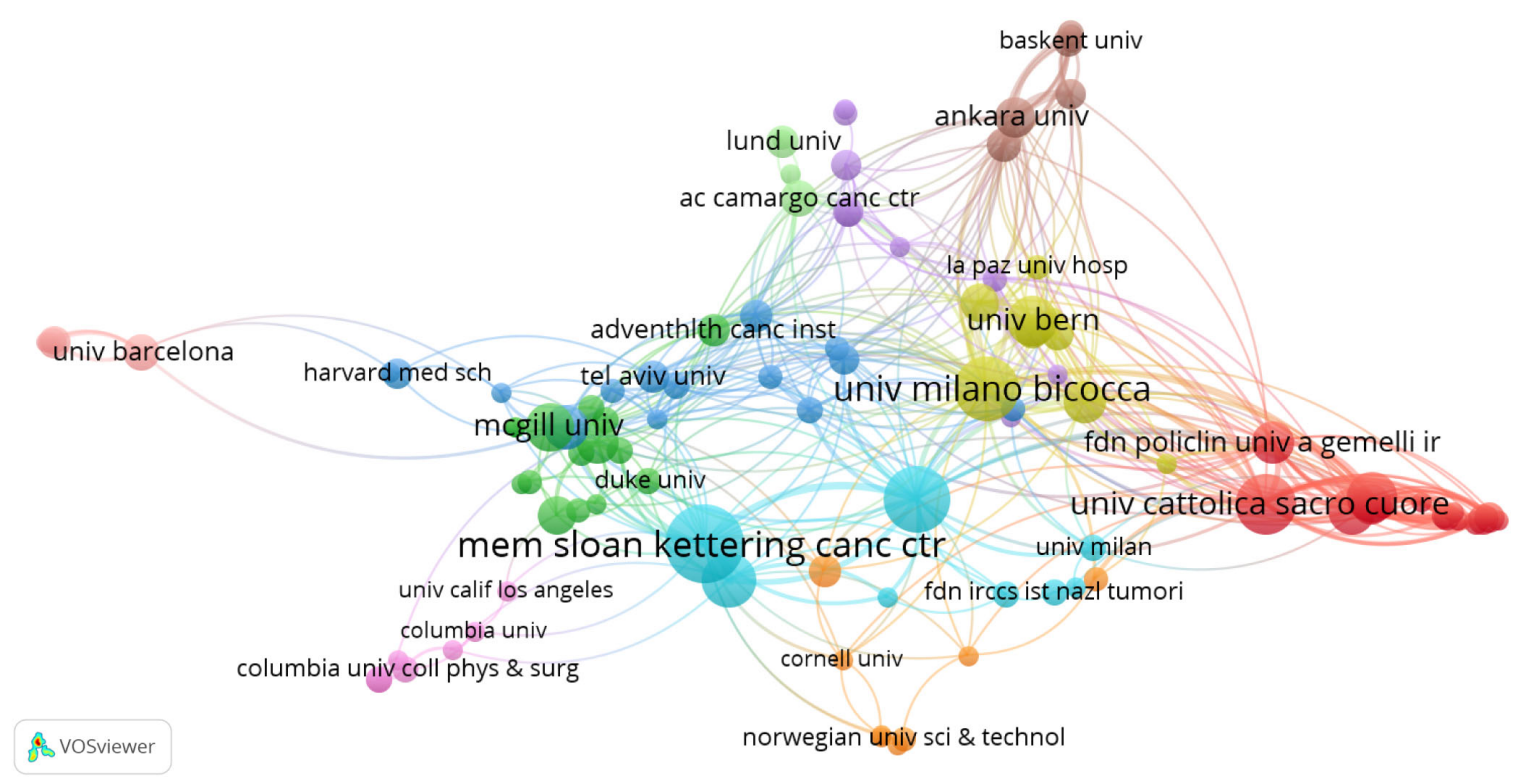
The cooperation networks between organizations.

A total of 841 institutions contributed to research in SLN mapping for endometrial cancer. The top 10 institutions with the highest publication counts are presented in **Table 2**, including four institutions each from the United States and Italy. Memorial Sloan Kettering Cancer Center ranked first, with 41 publications, an average citation frequency of 66.1, and an H-index of 29. While Weill Cornell Medicine produced fewer publications, 23 in total, it reported a notable average citation rate of 53.22. Both Memorial Sloan Kettering Cancer Center and University of Milano-Bicocca showed active collaboration with other institutions (**Figure 4**).

**Table 2:** Top 10 organizations of published articles.

Rank	Affiliation	Country	Publications	Total citation	Average citation	H-index
1	Mem Sloan Kettering Canc Ctr	USA	41	1260	66.1	29
2	Catholic University of the Sacred Heart	Italy	37	316	12.57	14
3	IRCCS Policlinico Gemelli	Italy	37	316	12.57	14
4	Mayo Clinic	USA	31	437	24.68	16
5	Univ Milano Bicocca	Italy	30	409	21.1	16
6	San Gerardo Hospital	Italy	25	382	23.32	16
7	Cornell University	USA	24	722	51.08	18
8	Assistance Publique Hopitaux Paris (APHP)	France	23	714	46.17	14
9	Weill Cornell Medicine	USA	23	721	53.22	18
10	Unicancer	France	22	676	40.68	13

### 3.4 Author analysis

**Figure 5:**
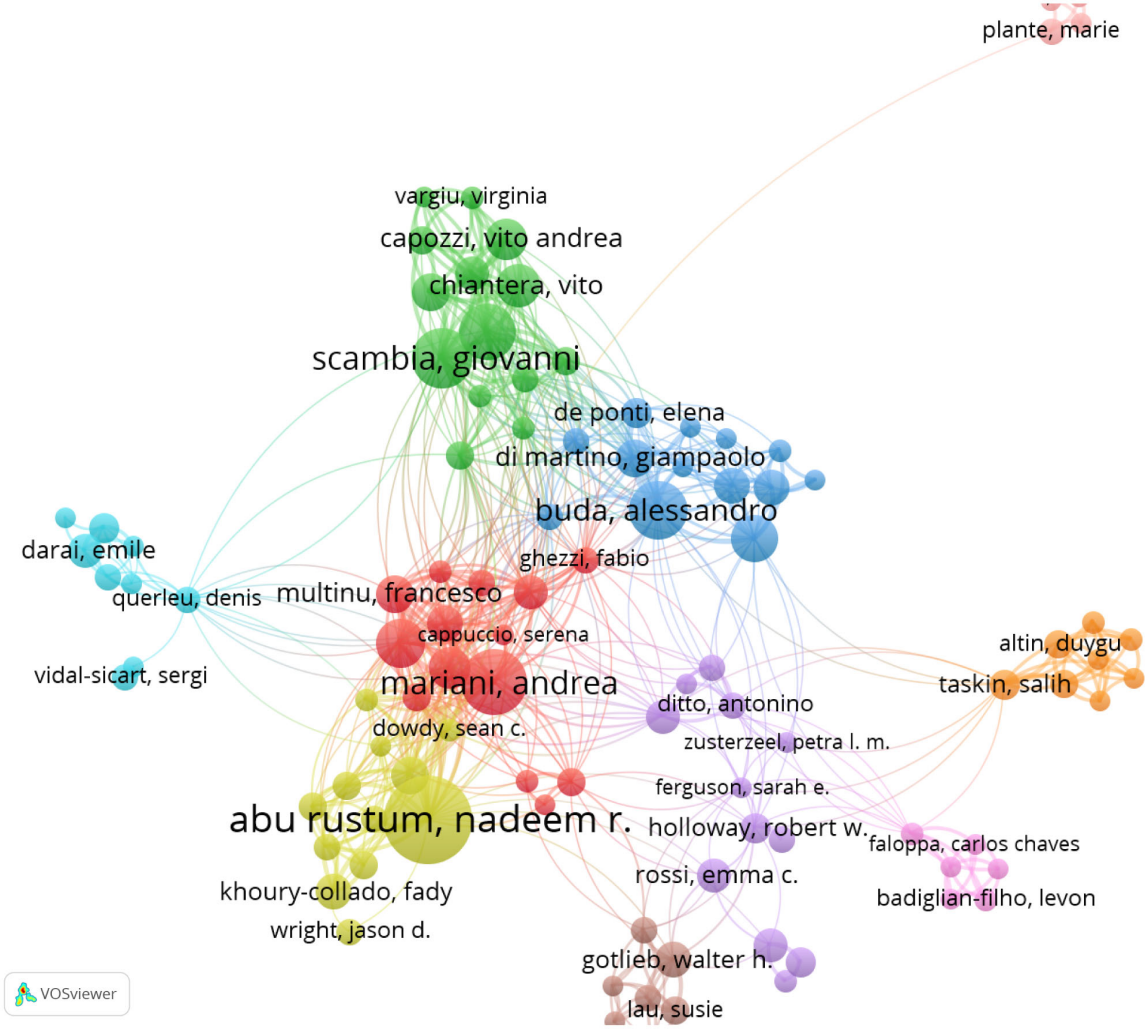
The cooperation networks between authors.

A total of 2,598 authors contributed to publications addressing the role of SLN in endometrial cancer. The top 10 most productive authors are listed in **Table 3**, with five based in the United States. Dr. Nadeem R. Abu-Rustum ranked first, having authored 35 articles with a total of 1,229 citations and an average citation count of 75.31 per article. **Figure 5** presents the co-authorship network among researchers who have published more than five articles. Notably, Dr. Nadeem R. Abu-Rustum, along with Marian Andrea, Alessandro Buda, and Giovanni Scambia, showed strong collaborative ties with other contributors in the field.

**Table 3:** Top 10 published article authors.

Rank	Author	Country	Documents	Citation	Average citation	H-index
1	Abu-Rustum, Nadeem, R	USA	35	1229	75.31	28
2	Mariani, Andrea	USA	28	419	26.68	16
3	Buda, Alessandro	Italy	27	406	23.04	16
4	Leitao, Mario M.	USA	22	695	58.05	17
5	Fanfani, Francesco	Italy	22	188	11.59	10
6	Scambia, Giovanni	Italy	19	215	15.84	11
7	Papadia, Andrea	Switzerland	18	341	29.72	12
8	Multinu, Francesco	USA	16	205	16.75	9
9	Weaver, Amy L.	USA	15	384	37.6	12
10	Di Martino, Giampaolo	Italy	14	231	23.5	10

### 3.5 Journal analysis

**Figure 6:**
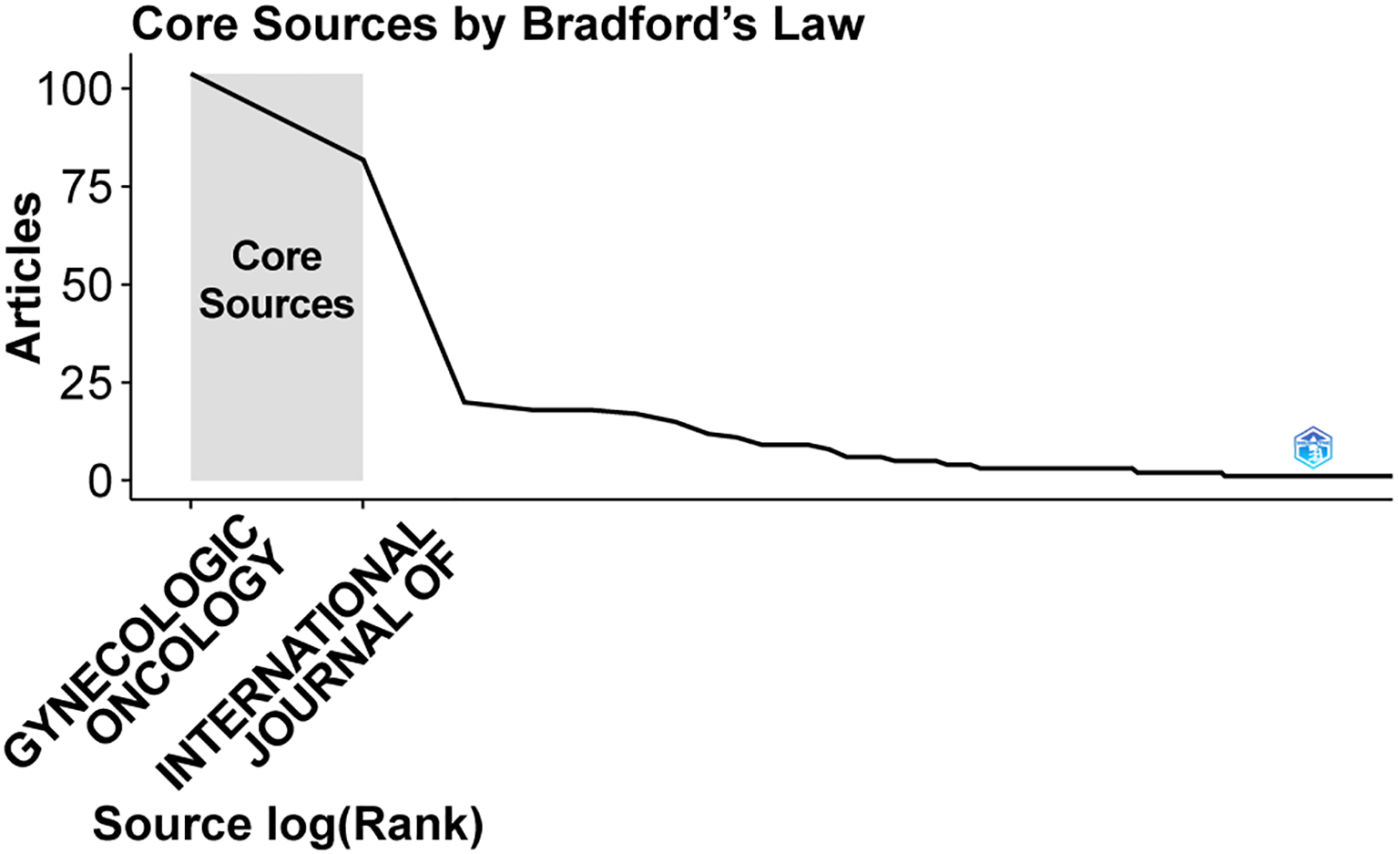
Core journals in which articles were published.

Altogether, 125 journals have published work on SLN in endometrial cancer. *Gynecologic Oncology* led the field with 104 articles, amassing 1,464 citations and an average citation frequency of 50.81 (**Table 4**). The *International Journal of Gynecological Cancer* and the *Journal of Minimally Invasive Gynecology* followed with 82 and 20 articles, respectively. Other journals published comparatively fewer papers on the topic. According to Bradford’s Law, as shown in **Figure 6**, *Gynecologic Oncology* and the *International Journal of Gynecological Cancer* were identified as the core journals in this area, reflecting their central role in disseminating research on SLN in endometrial cancer.

**Table 4:** Top 10 contributions of different journals.

Rank	Journals	Publications	Citations	Average citations	IF&JCR division (2022)
1	GYNECOLOGIC ONCOLOGY	104	1646	50.81	4.7Q 1
2	INTERNATIONAL JOURNAL OF GYNECOLOGICAL CANCER	82	835	17.44	4.8Q 1
3	JOURNAL OF MINIMALLY INVASIVE GYNECOLOGY	20	270	15.9	4.1Q1
4	ANNALS OF SURGICAL ONCOLOGY	18	498	37.56	3.7Q1
5	EUROPEAN JOURNAL OF GYNECOLOGICAL ONCOLOGY	18	176	11.78	2.6Q3
6	JOURNAL OF GYNECOLOGIC ONCOLOGY	17	234	14.41	3.9Q1
7	CANCERS	12	45	3.92	5.2Q2
8	EUROPEAN JOURNAL OF OBSTETRICS GYNECOLOGY AND REPRODUCTIVE BIOLOGY	11	40	3.91	2.6Q3
9	ARCHIVES OF GYNECOLOFY AND OBSTERTRICS	9	82	9.89	2.6Q3
10	EJSO	9	122	14.44	3.8Q1

### 3.6 Keyword analysis

**Figure 7:**
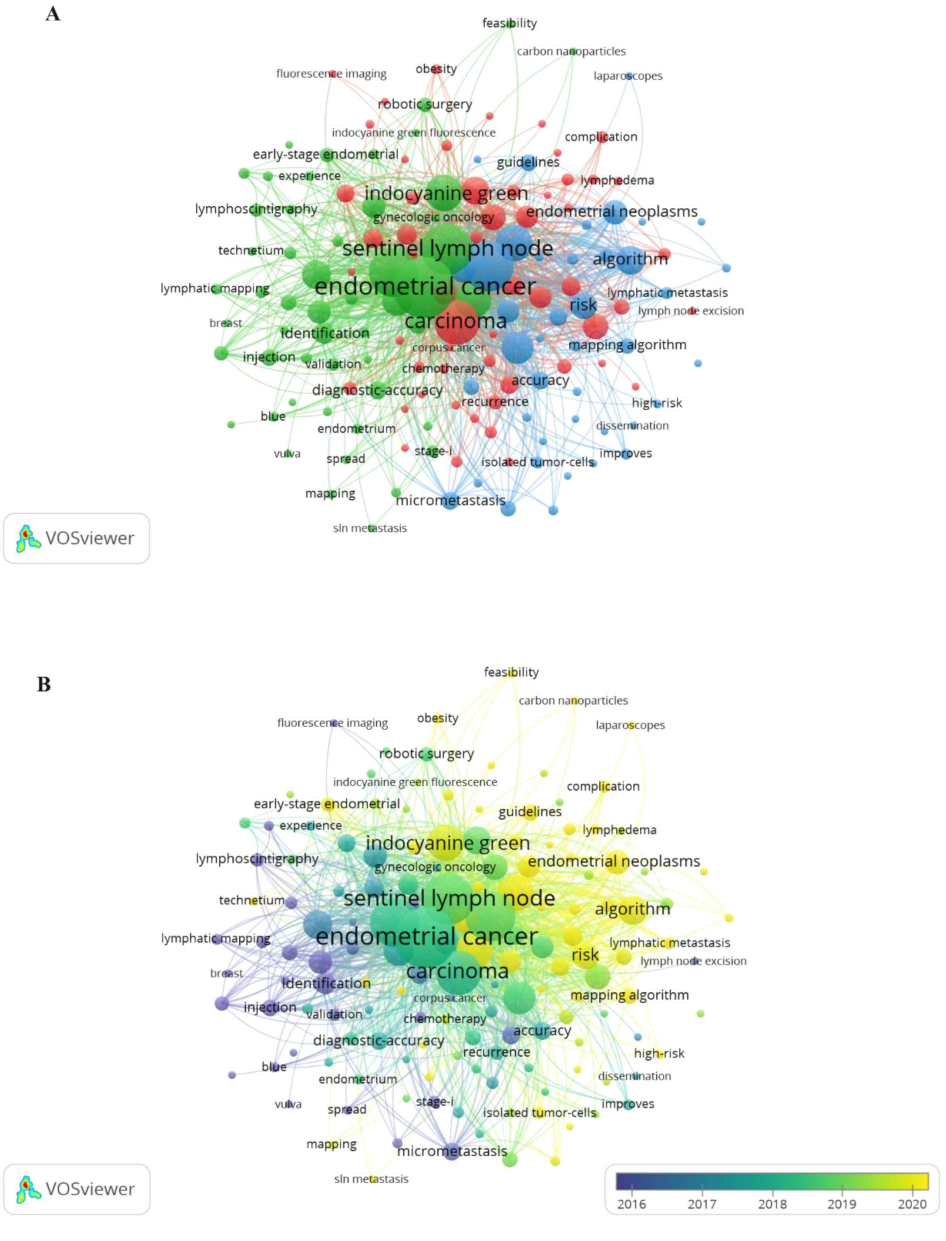
**(A)** Keyword networks appeared greater than or equal to 6. **(B)** Overlay networks of keywords.

Following manual consolidation of synonymous terms, a total of 1,080 distinct keywords were identified. **Table 5** lists the top 10 most frequently occurring keywords. As shown in **Figure 7A**, 148 keywords appeared at least six times across the analyzed articles and were grouped into three main clusters. The largest cluster, marked in red, included 55 keywords mainly related to the surgical application of SLN in gynecologic oncology. Terms in this group included carcinoma, lymph node excision, chemotherapy, recurrence, complication, and lymphedema. The second-largest cluster, shown in green, consisted of 52 keywords centered on the use of fluorescent dye in lymphatic mapping for gynecologic oncology. Representative keywords included endometrial cancer, sentinel lymph node, indocyanine green, lymphoscintigraphy, blue dye, mapping, and injection. The third cluster, shown in blue, included 41 keywords and focused on SLN management in gynecologic oncology, featuring terms such as endometrial neoplasms, lymphatic metastasis, risk, accuracy, and guidelines. Across all clusters, frequently recurring keywords revolved around central themes like endometrial cancer, sentinel lymph node, lymphadenectomy, biopsy, and indocyanine green (ICG). **Figure 7B** a temporal overlay in which node colors represent the average time of keyword appearance. According to the color scale in the lower right, earlier terms are shown in purple, while more recent ones trend toward yellow. The progression of SLN research initially emphasized cervical cancer, vulvar cancer, and breast cancer, and later shifted toward endometrial cancer and tumor metastasis. Current topics of interest have concentrated on areas such as complication, guidelines, outcomes, and survival.

**Table 5:** Top 10 keywords of published articles.

Rank	Keyword	Occurrences	Total link strength
1	Endometrial cancer	376	3014
2	Sentinel lymph node	265	2076
3	Lymphadenectomy	252	2120
4	Biopsy	244	2076
5	Carcinoma	203	1819
6	Indocyanine green	139	1233
7	Multicenter	136	1254
8	Trial	135	1132
9	Metastasis	103	915
10	Women	93	811

## 4 Discussion

### 4.1 Summary of main results

Sentinel lymph nodes have become a central focus in surgical strategies, especially for managing early-stage endometrial cancer in recent years. This study analyzed 545 articles related to SLN in the context of endometrial cancer and systematically identified research hotspots and emerging themes through bibliometric analysis.

Two software tools, VOSviewer and the R package “bibliometrix,” were used to map the research landscape, highlighting leading countries or regions, institutions, authors, journals, and keyword patterns in this area.

A steady rise in SLN-related publications has been evident since 2002, with annual output exceeding 30 articles by 2017. The United States led in both the number of publications and total citations, followed closely by Italy. Among institutions, Memorial Sloan Kettering Cancer Center was the most productive and received the highest citation frequency. The most productive and cited researchers in the field included Abu-Rustum Nadeem R, Mariani Andrea, Buda Alessandro, Leitao Mario M, and Fanfani Francesco. The top journals by citation and volume were *Gynecologic Oncology* and the *International Journal of Gynecological Cancer*. Keyword co-occurrence analysis revealed three distinct clusters, reflecting a structured development of research themes and marking the trajectory of scholarly interest in SLN use for endometrial cancer over time.

### 4.2 The role of sentinel lymph node in endometrial cancer

At present, commonly used techniques for sentinel lymph node (SLN) mapping include isosulfan blue, methylene blue, indocyanine green (ICG), and radiolabeled colloidal technetium-99 (Tc99) (29). ICG is a water-soluble tricarbocyanine dye that emits fluorescence in the near-infrared (NIR) spectrum and is approved by the FDA for vascular and hepatobiliary imaging (29). Optimal detection rates are achieved when ICG is diluted to 0.5–1.25 mg/mL with sterile water, and 2–4 mL is injected (30, 31). ICG-guided mapping can be applied in open, laparoscopic, or robotic surgeries and offers better performance than blue dyes, particularly in obese patients (32). Different injection sites, such as the cervix, corpus uteri, and utero-ovarian ligaments, often yield the same SLN, supporting the flexibility of injection approaches (33).

In endometrial cancer, lymph node metastasis is the most frequent form of extrauterine dissemination. Lymph node assessment is a fundamental component of surgical management for early-stage disease and is both a strong prognostic indicator and a reliable predictor of recurrence (34). However, only up to 10% of patients with early-stage endometrial cancer present with nodal metastasis (35). The FIRES trial, which evaluated 385 patients with stage I endometrial cancer, reported an SLN detection rate of 86% and a negative predictive value of 99.6% (15). Comparative studies examining three lymphadenectomy strategies, systematic dissection, selective dissection, and SLN mapping, have found SLN mapping to be significantly more effective in patients with low-risk disease (36). One such study assessed both cost and quality of life three years postoperatively: systematic lymphadenectomy incurred costs of $18,041 with an effectiveness of 2.79 quality-adjusted life years (QALYs), elective lymph node dissection cost $17,036 with 2.81 QALYs, while SLN excision had the lowest cost at $16,401 and the highest quality of life at 2.87 QALYs (36). In a study by Ducie et al., involving patients with intermediate- or high-risk factors for advanced endometrial cancer, 202 individuals who underwent SLN resection showed similar detection rates to 210 who had systematic lymph node resection (*p*=0.76, *p*=0.23) (37). Additionally, Barbara Geppert et al. studied 188 patients and found that SLN mapping resulted in a shorter operative time (91 minutes) and a significantly lower incidence of postoperative lymphedema, 1.3% versus 18.1% (*p*=0.0003) (38).

Recent research has increasingly focused on prognosis and survival outcomes in patients undergoing SLN mapping for endometrial cancer. A comparative study conducted by two Italian institutions found no significant differences in disease-free survival among patients who underwent SLN mapping, systemic lymph node dissection, or a combination of both procedures (39). Capozzi and colleagues examined long-term survival in high-risk patients who received either SLN biopsy alone or systematic pelvic lymph node dissection. Their findings revealed no significant difference in disease-free survival (*p* = 0.74) or overall survival (*p* = 0.62) between the two groups. Among patients with nodal metastasis, overall survival (*p* = 0.43) and disease-free survival (*p* = 0.46) were also similar between the two treatment approaches (40). Cuccu Ilaria et al. evaluated 5-year outcomes following SLN mapping in patients with high-intermediate and high-risk endometrial cancer (41). The study included 242 patients and found that, compared with lymphadenectomy, SLN mapping had no significant impact on 5-year disease-free survival (HR: 1.233; 95% CI: 0.6217 to 2.444; *p* = 0.547, log-rank test) or overall survival (HR: 1.505; 95% CI: 0.6752 to 3.355; *p* = 0.256, log-rank test). SLN mapping did not increase the risk of lymph node recurrence in women with high-intermediate or high-risk disease. Additionally, no significant difference in surgical complications at 30 days was observed between the SLN mapping group and the lymphadenectomy group (1.7% *vs*. 11.9%, *p* = 0.079) (41). Ongoing prospective studies are examining the role of SLN in relation to prognosis in endometrial cancer. The SELECT trial is investigating pelvic and nonvaginal recurrence in women with sentinel node-negative intermediate-risk disease (42), while the ENDO-3 trial is focused on postoperative complications and 4.5-year disease-free survival in patients undergoing SLN resection (43). There are indeed many clinical studies on the role of sentinel lymph nodes in the survival of endometrial cancer, which is consistent with the results of this study. The keyword network of this study shows that in recent years, sentinel lymph node research has been dedicated to survival, occurrence, guidance, lymph node metastasis, etc. This indicates that bibliometrics can provide reference directions for the future research development trends and hotspots.

Pathological ultrastaging is a key component of the sentinel lymph node (SLN) algorithm. It involves additional serial sectioning of SLNs stained with hematoxylin and eosin, with or without the use of immunohistochemical analysis (44). Ultrastaging allows for the identification of lesions that would be missed during standard pathological evaluation. It significantly improves the detection of low-volume disease, increasing the identification of micrometastases (lesions >0.2 mm and ≤2 mm) by over 50% and isolated tumor cells (single cells or clusters ≤0.2 mm) by nearly 100% (45). A prognostic study involving 494 low-risk endometrial cancer patients with isolated tumor cell metastasis in SLNs found that, in the absence of adjuvant therapy, those with isolated tumor cells had a lower recurrence-free survival rate (*p* < 0.01), though overall survival was unaffected (46).

Molecular subtyping, regardless of histologic type, also plays a critical role in determining prognosis in endometrial cancer (47). A study involving 237 patients revealed that occult lymph node metastasis occurred more frequently in the MSI group than in the MSS group (19% *vs*. 6.7%, *p* = 0.005), identifying MSI as an independent risk factor for occult nodal involvement in early-stage disease (48). Looking ahead, integrating SLN visualization with molecular classification may improve the precision of prognostic assessments. The introduction of novel tracers holds potential for increasing the accuracy and consistency of SLN imaging. Furthermore, applying big data analytics and artificial intelligence to SLN imaging may lead to more precise prognostic models. Matteo Pavone et al. demonstrated the feasibility of using artificial intelligence algorithms to analyze SLN dissection videos, marking a step toward the broader use of AI in this field (49).

This study does have some limitations. First, data were sourced solely from the Web of Science Core Collection, excluding potentially relevant publications from other databases. Second, the analysis was limited to English-language articles, which may have led to the omission of valuable studies published in other languages. Lastly, recently published articles may not have been fully represented in the citation analysis due to the short time frame available for citation accumulation.

## 5 Conclusions

This bibliometric study offers a historical and analytical overview of research on the role of SLNs in endometrial cancer. The volume of related publications has steadily increased over the past decades, reaching a peak in 2021. This analysis identified the most active countries or regions, institutions, authors, and journals, and highlighted key research trends, particularly the association between SLNs and prognosis, recurrence, and treatment strategies in endometrial cancer. The findings emphasize the importance of strengthening collaboration among North America, Europe, and China to advance research in this field. By mapping the current landscape, this study helps researchers better understand the evolving role of SLNs in endometrial cancer treatment and may also serve as a reference for institutional planning and policy development.

## Data Availability

The raw data supporting the conclusions of this article will be made available by the authors, without undue reservation.
